# Analysis of Nano-ZnO-Modified Asphalt Compatibility Based on Molecular Dynamics

**DOI:** 10.3390/ma16134710

**Published:** 2023-06-29

**Authors:** Yunlan Xie, Pandeng Yu, Ming Zhai

**Affiliations:** School of Mechanics and Safety Engineering, Zhengzhou University, Zhengzhou 450001, China; 202012252014781@gs.zzu.edu.cn (Y.X.); 202012252014786@gs.zzu.edu.cn (P.Y.)

**Keywords:** nano-ZnO, modified asphalt, molecular dynamics, compatibility

## Abstract

Nano-ZnO has a large specific surface area, small particle size, and strong polarity and can be used as an additive to modify the base asphalt. In this paper, the compatibility mechanism between nano-ZnO modifier and asphalt is analyzed. Solubility parameters, interaction energNano-ZnO and mean square displacement of nano-ZnO in matrix asphalt were calculated at different temperatures to study the compatibility of the nano-ZnO modifier and the matrix asphalt. The radial distribution functions and radii of gyration of the asphalt’s four components under the action of the nano-ZnO additive were calculated to investigate the effect of nano-ZnO on the molecular structure of the asphalt. The results show that the best compatibility between nano-ZnO and matrix asphalt is observed at 150 °C, especially when the nano-ZnO particle size was 6 Å. The particle sizes of nano-ZnO have little effect on the temperature at which the nano-ZnO-modified asphalt achieved its highest structural stability. Around 150 °C, the nano-ZnO-modified asphalt system with different particle sizes exhibit the highest stability and best compatibility. The addition of nano-ZnO improves the compactness of the asphalt structure and makes the asphalt more stable.

## 1. Introduction

Asphalt has excellent waterproofing and viscoelastic properties and is a common pavement material used in road construction [1]. Although asphalt pavements have good compressive, tensile, and flexural resistance, prolonged exposure to harsh climatic conditions and a variety of factors can significantly degrade their performance, causing surface cracks or more serious damage to the pavement [2], reducing the smoothness of the pavement and destroying the integrity of the structure, which in turn affects the quality of vehicle movement and seriously affects the performance and service life of the pavement [3]. Damaged or failing asphalt pavements not only affect driving safety and comfort but can also lead to a vicious cycle of distress [4,5], resulting in traffic accidents and property damage. Therefore, it is important to study the performance of asphalt in pavement safety and to improve it.

Nanomaterials have a huge specific surface area and special surface properties that can be used to improve the properties of bitumen. Nano-ZnO not only has the properties of general nanomaterials but also has excellent absorption and shielding effects on UV light. More and more scholars use nano-ZnO as an additive to modify matrix asphalt and reveal the modification mechanism of nanomaterials. This provides solutions and a scientific basis for studying asphalt compatibility, reducing asphalt pavement diseases, and improving pavement service life and durability [6]. In recent years, many new nanomaterials, such as ZnO [7,8], TiO_2_ [9,10], CaCO_3_ [11], SiO_2_ [12], and CeO_2_ [13], have been widely used in asphalt to enhance specific properties of asphalt. Zhu [14] investigated the road performance of nano ZnO-modified asphalt. The results showed that nano-ZnO both physically and chemically modified the asphalt and that they improved the high-temperature rutting resistance and viscosity–temperature properties of the asphalt. Zhang [15] studied the UV aging resistance of asphalt and found that the addition of nano-ZnO particles significantly improved the UV aging resistance of asphalt. Chen et al. [16] prepared a nano-ZnO modifier by the high-speed shear method in the laboratory and added it to asphalt to study the performance of asphalt. The results show that the nano ZnO-modified asphalt significantly improved road performance. The optimum dosage of nano-ZnO was 4%, and the performance of asphalt was the best. Liu et al. [17] studied the aging resistance of asphalt in indoor experiments and found that after the surface modification treatment, nano-ZnO could be evenly coated on the asphalt, thus greatly enhancing the aging resistance of the asphalt. Currently, nano-ZnO is receiving more and more attention due to its good properties and promising applications, and it has already been produced on a large scale for industrial use. Using nano-ZnO as an additive is an effective way to improve various properties of asphalt.

The compatibility of the modifier is an important factor in determining the effectiveness of asphalt modification, which reflects the performance of the modified asphalt and enables the modifier to form a good bond with the asphalt. Wang et al. [18] constructed a molecular model of bitumen and carbon nanotubes and characterized the interactions in each molecular system using bonding and non-bonding energies, and the results showed that carbon nanotubes increased the roughness of the interface and the pull-out behavior of carbon nanotubes alleviated interfacial failure and also enhanced the degree of compatibility between polymer and bitumen. Wang et al. [19,20] constructed a model of rubber-modified bitumen and performed molecular dynamics simulations at different temperatures to calculate the solubility parameters and molecular potential energy of both. The results showed that the best compatibility between asphalt and rubber powder was achieved at 160 °C and was taken for experimental verification, providing strong support for the application of molecular dynamics techniques in the compatibility study of asphalt and modifiers. Su et al. [21,22] found that SBS modifier, nano-ZnO modifier, and nano-ZnO/SBS modifier of different sizes could improve the mechanical properties of the matrix asphalt, significantly increasing the elastic modulus, shear modulus, and bulk modulus of the matrix asphalt, with the nano-ZnO/SBS modifier being particularly effective. Guo et al. [23,24] studied the compatibility of matrix asphalt and three rubber-modified asphalts by molecular dynamics simulations. It was found that solubility parameters and binding energy were effective indicators for assessing the compatibility of asphalts, and the optimum admixture of modifiers in the three rubber-modified asphalts was obtained. Yu et al. [25] studied the compatibility of epoxy resin and matrix asphalt by using solubility parameters and interaction energy as evaluation indicators; the compatibility of the bitumen was studied, and it was shown that the addition of epoxidized soybean oil to the cold mixed epoxy bitumen could significantly improve the compatibility of the modified bitumen system.

The above studies on the modification mechanism of asphalt by nano-ZnO are not comprehensive, and only some influencing factors are analyzed. To reveal the compatibility mechanism between nano-ZnO modifier and asphalt, AAA-1 asphalt was used as the research object. Based on molecular dynamics (MD) technology and Materials Studio (MS) software, the matrix asphalt model, the nano-ZnO model, and the nano-ZnO-modified asphalt model were established. The diffusion properties of nano-ZnO in matrix asphalt and the interaction between nano-ZnO and matrix asphalt molecules were studied. The radial distribution function and rotation radius of the nano-ZnO/matrix asphalt model at different temperatures were calculated, and the clustering and adsorption behaviors between the modifier and the asphalt were analyzed. The effect of nano-ZnO on the molecular structure of asphalt and the compatibility mechanism between nano-ZnO modifier and asphalt were investigated.

## 2. Simulation Methodology

### 2.1. Establish a Molecular Model of Matrix Asphalt

**Figure 1:**
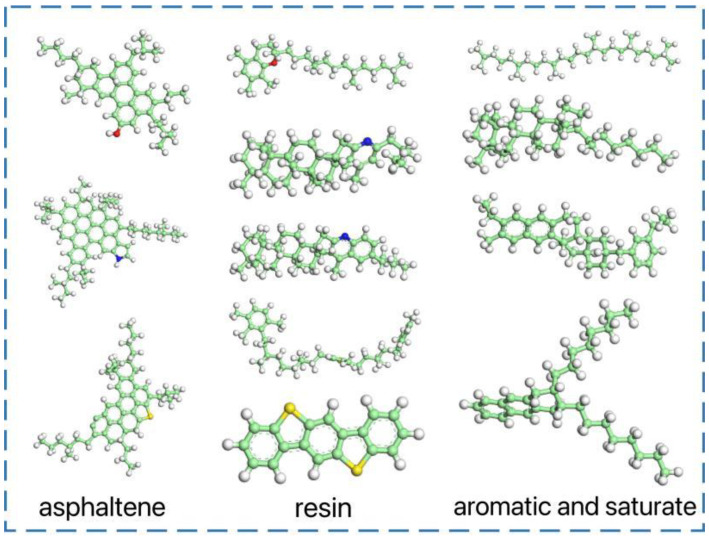
Four components model for asphalt (Carbon atoms are green, hydrogen are white, oxygen are red, nitrogen are blue, sulfur are yellow).

**Figure 2:**
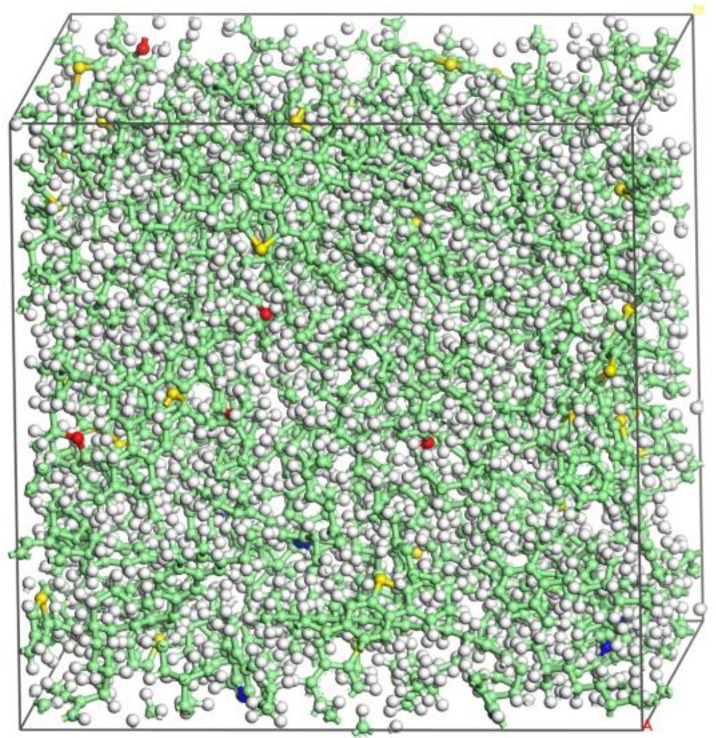
Molecular model of matrix asphalt (Carbon: green, hydrogen: white, oxygen: red, nitrogen: blue, sulfur: yellow).

Three standardized asphalt samples [26], AAA-1, AAK-1, and AAM-1, were proposed. In this paper, AAA-1 was used to represent the matrix asphalt and 12 molecules were used to characterize the four components of bitumen. Molecular models of the four components of asphalt were constructed using Materials Studio [27] (MS) software according to the ratios of C, H, O, N, and S elements in the samples, as shown in Figure 1. The 12 molecular models were loaded into the Amorphous modulus module and combined in an amorphous 3D periodic boundary progenitor cell to create a molecular model of the matrix asphalt, as shown in Figure 2.

**Figure 3:**
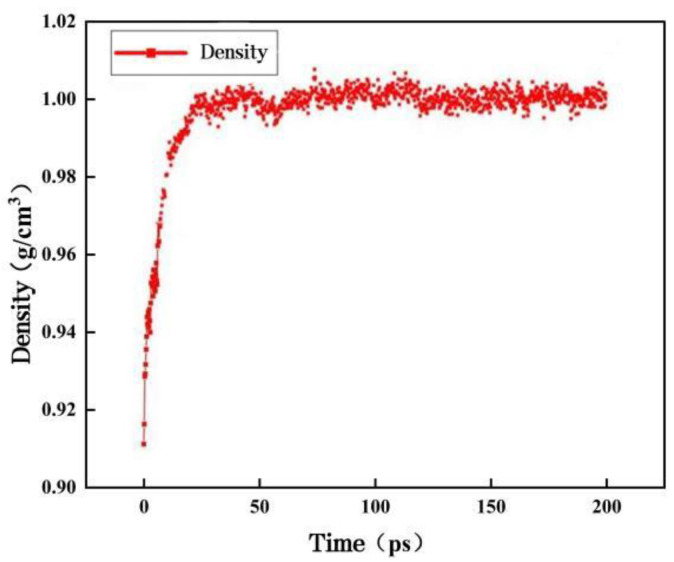
Density of the matrix asphalt model.

After establishing the molecular model of the matrix asphalt, molecular dynamics calculations were performed using the Forcite module to make it equilibrium, and the COMPASS II force field was used to reduce the energy of the system using the most rapid descent method to minimize its potential energy. After optimizing and minimizing the energy of the system, it was relaxed for 200 ps under the NPT system with a pressure of 1 atm and a temperature of 298.15 K to obtain a stable bitumen model with dimensions of 37.75 Å × 37.75 Å × 37.75 Å. Figure 3 shows the density of the matrix bitumen model.

As can be seen from Figure 3, the density of the matrix asphalt model is approximately 1.006 g/cm^3^, which is very close to the laboratory-measured density of 1.0 g/cm^3^, and the model can be judged to be reasonable [28].

### 2.2. Molecular Modeling of Nano-ZnO Molecules

**Figure 4:**
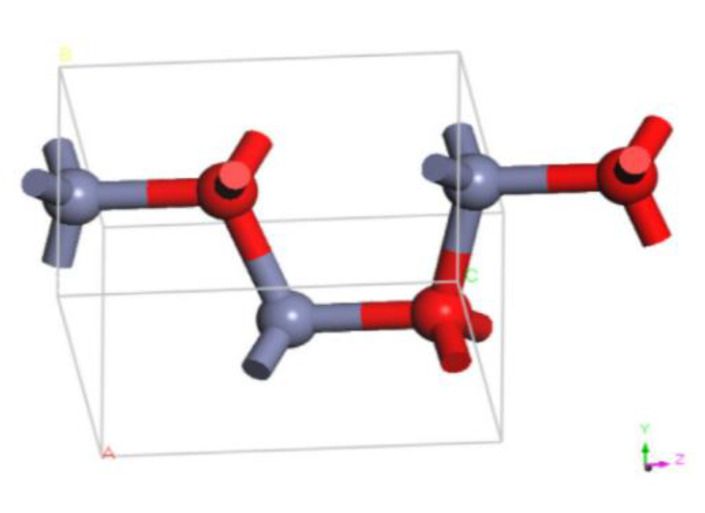
Crystal model of ZnO.

**Figure 5:**
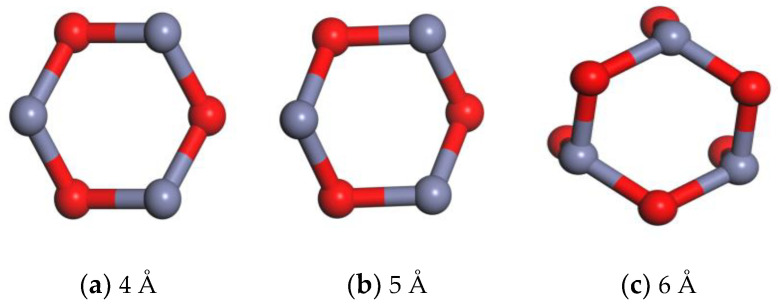
ZnO clusters of different diameters.

Based on the lattice parameters and space group information, the crystal interface model was reconstructed using MS software to construct the nano-ZnO crystal model (Figure 4). In the MS cluster construction interface, the clusters were set to be spherical, and the cluster diameters were 4 Å, 5 Å, and 6 Å to build three diameters of nano-ZnO clusters, and their 3D models are shown in Figure 5.

### 2.3. Molecular Modeling of Nano-ZnO-modified Asphalt

**Figure 6:**
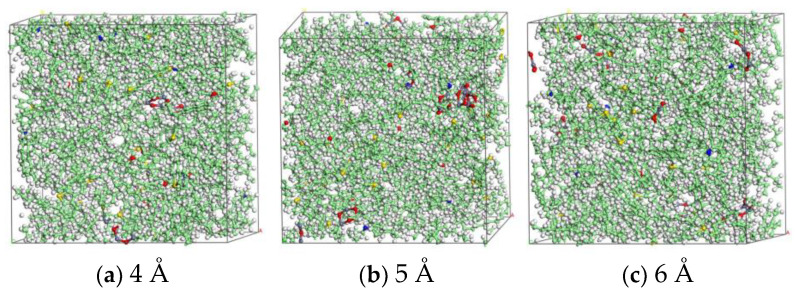
Molecular models of nano-ZnO-modified bitumen with different nano-ZnO diameters (Carbon: green, hydrogen: white, oxygen: red, nitrogen: blue, sulfur: yellow, zinc: grey).

According to the information on the composition of nano-ZnO in the nano-ZnO-modified bitumen model given in Table 1, nano-ZnO was incorporated into the AAA-1 matrix bitumen model to obtain three nano-ZnO-modified bitumen models with diameters of 4 Å, 5 Å and 6 Å, where the nano-ZnO incorporation amount was all about 4.3% (Table 1). The three nano-ZnO-modified bitumen models were geometrically optimized using the fastest descent method for 5000 iterations to achieve the lowest potential energy. The three nano-ZnO-modified bitumen models were relaxed for 200 ps each in an isothermal isobaric (NPT) system at 1 atm, 298.15 K. After the energy, density, and temperature of the system were stabilized, the nano-ZnO-modified bitumen molecular models were obtained, as shown in Figure 6.

**Figure 7:**
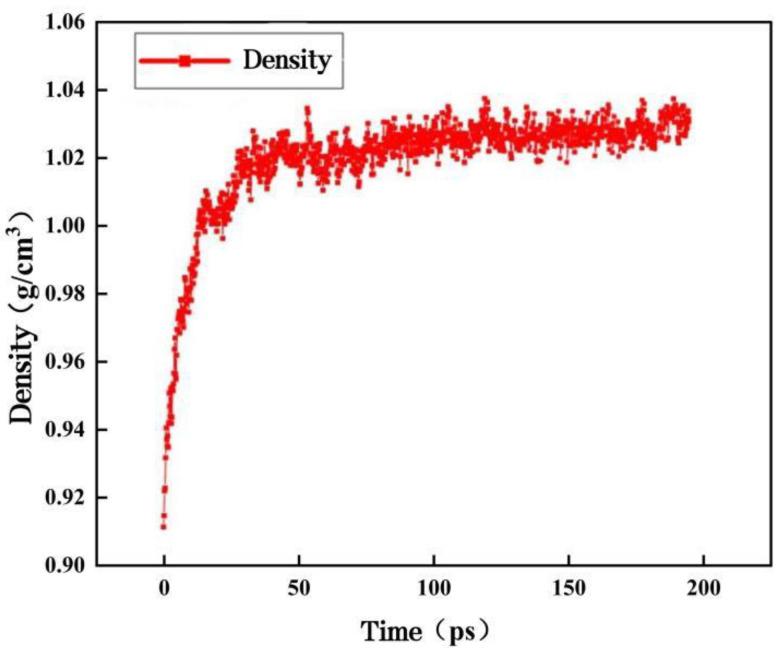
4 Å nano-ZnO-modified asphalt density variation graph.

Figure 7 gives a graph of the density variation of the 4 Å nano-ZnO-modified bitumen. When equilibrium is reached, the side length of the model is 37.95 Å, and the density is approximately 1.035 g/cm^3^, which is closer to the laboratory-measured density of 1.04 g/cm^3^, and the model is reasonable. After verification by the same method, the constructed nano-modified bitumen models with ZnO particle sizes of 5 Å and 6 Å are also reasonable.

### 2.4. Molecular Dynamics Calculation of Solubility Parameters

Solubility parameters are used to characterize the compatibility between different materials, especially non-polar materials. If the solubility parameters of two materials are similar, they can be mixed well together. The solubility parameter was used to evaluate the compatibility of ZnO nanoparticles with asphalt. According to the heat of mixing theory of polymer blends, the binding energy density is the energy required to remove all intermolecular forces from 1 mol of material, it is a physical quantity characterizing intermolecular interactions, and the square root of the binding energy density is the solubility parameter. Molecular dynamics simulations were used to calculate the cohesion energy density for the asphalt system and the ZnO system to obtain the solubility parameter [29], as shown in Equations (1) and (2).

Solubility parameters can be used to assess the compatibility of various materials, especially non-polar materials. When the difference between the solubility parameters of two substances is small, they are able to combine more effectively, thus enhancing the thermodynamic stability and compatibility of the system. The better the compatibility between the substances, the higher the stability of the mixed system. The solubility parameter of a substance is closely related to the cohesion energy density. The cohesion energy density is the energy of interaction between all molecules within 1 mol of a substance and is used to characterize intermolecular interactions, the square root of which determines the solubility parameter of the substance. Through molecular dynamics simulation, the cohesion energy density of matrix asphalt and nano-ZnO-modified asphalt can be calculated to determine their solubility parameters and evaluate the compatibility of nano-ZnO materials with matrix asphalt.

(1) $$CED=\frac{coh}{V}$$

(2) $$\delta =\sqrt{CED}$$

where *CED* is cohesion energy density, J/cm^3^; *coh* is binding energy, J; δ is solubility parameter, (J/cm^3^)^1/2^.

### 2.5. Interaction Energy

The compatibility of a substance is closely related to the interactions between the molecules that make up the substance. In order to further investigate the mechanism of compatibility changes in nano-modified asphalt, the interaction energy between molecules of asphalt materials also needs to be analyzed. The interaction energy can be used to measure the strength of intermolecular interactions, predict the mixing ability and compatibility of different materials in a mixture, and evaluate the stability of the system. The interaction energy of nano-ZnO particles with bitumen is non-bonding energy, which mainly consists of van der Waals energy and electrostatic energy. In the (COMPASS)II force field, the non-bonding energy, van der Waals energy, and electrostatic energy are calculated for the two systems a,b as an example:

(3) $$E_{n}=E_{abn}-E_{an}-E_{bn}$$

(4) $$E_{V}=E_{abV}-E_{aV}-E_{bV}$$

(5) $$E_{\varepsilon }=E_{ab\varepsilon }-E_{a\varepsilon }-E_{b\varepsilon }$$

where $E_{n}$ is the Non-bond energy interaction energy (Kcal/mol) of the a,b system; $E_{V}$ is the Van der Waals energy interaction energy (Kcal/mol) of the a,b system; $E_{\varepsilon }$ is electrostatic interaction energy (Kcal/mol) of the a,b system; $E_{abn}$, $E_{an}$, and $E_{bn}$ is Non-bonding energy (Kcal/mol) of the ab co-mingled system, system a, system b; $E_{abV}$, $E_{aV}$, and $E_{bV}$ is Van der Waals potential energy (Kcal/mol) of the ab co-mingled system, system a, system b; $E_{ab\varepsilon }$, $E_{a\varepsilon }$, and $E_{b\varepsilon }$ is electrostatic potential energy (Kcal/mol) of the ab co-mingled system, system a, and system b.

### 2.6. Mean Square Displacement

The diffusion coefficient is an important measure of the ability of a substance to propagate under different concentration conditions, defined as the rate of passage of a mass of material per unit area per unit concentration gradient, and can be used to represent the rate of molecular movement in asphalt systems [30]. In molecular dynamics analysis, the diffusion coefficient of a substance can be determined by calculating the mean square displacement (MSD) curve of the system. The MSD is calculated by the formula:

(6) $$MSD(t)=\langle |r_{i}(t)-r_{i}(0){|}^{2}\rangle$$

In Equation (6), $r_{i}(0)$ is the position of particle *i* at the initial moment. $r_{i}(t)$ is the position of particle *i* at moment.

Once the mean square displacement (MSD) of the system has been obtained, the diffusion coefficient of the system can then be calculated from Equation (7):

(7) $$D=\frac{1}{6N}\underset{t\to \infty }{\lim}\frac{d}{dt}\sum_{i=1}^{N}\langle |r_{i}(t)-r_{i}(0){|}^{2}\rangle$$

In Equation (7), *D* denotes the diffusion coefficient of the system; *N* denotes the number of molecules in the system; the differential term is used to describe the linear relationship between the mean square displacement and time. As the number of molecules in the system is fixed, the diffusion coefficient is linearly related to the slope of the MSD curve. The larger the slope of the MSD curve, the larger the diffusion coefficient and the stronger the molecular diffusion ability. As the simulation is performed in a finite time, the diffusion coefficient can be approximated in the actual calculation as follows:

(8) $$D=\frac{a}{6}$$

where *a* is the linear slope of the mean square displacement versus time.

### 2.7. Radial Distribution Function

The radial distribution function (RDF) reflects the relative density between different molecules in a given range, and it can accurately reflect the state of distribution of molecules in a given environment [31]. *g*(*r*) is commonly used to represent the probability of certain molecules appearing at a distance r from each other in an asphalt system, expressed as numerous separated spikes, and it can characterize the agglomeration behavior in real asphalt, reflecting the aggregation situation of molecules within the asphalt system. The radial distribution function can be calculated from Equation (9).

(9) $$g(r)=\frac{1}{4\rho \pi r^{2}\xi r}\frac{\sum_{t=1}^{T}\sum_{j=1}^{N}\Delta N(r\to r+\xi r)}{N\times T}$$

where *N* is the total number of molecules; *T* is the total calculation time (ps); *r* is the distance from the reference molecule; ΔN is the number of molecules in the interval of the system; ρ is the density of the system.

### 2.8. Radius of Gyration

The radius of gyration (*R_g_*) can be used to measure the structure of a polymer material. It reflects the internal diameter of the material as it moves around the center of mass and describes the size of the molecular volume, the change in structure, and the closeness of the polymers to each other as they move. The radius of rotation can be expressed as

(10) $$R_{g}={\left(\frac{\sum r^{2}m}{\sum m}\right)}^{\frac{1}{2}}$$

where *r* is the distance from the center of mass; *m* is the mass of the molecular branch chain.

## 3. Results and Discussion

### 3.1. Solubility Parameters

**Figure 8:**
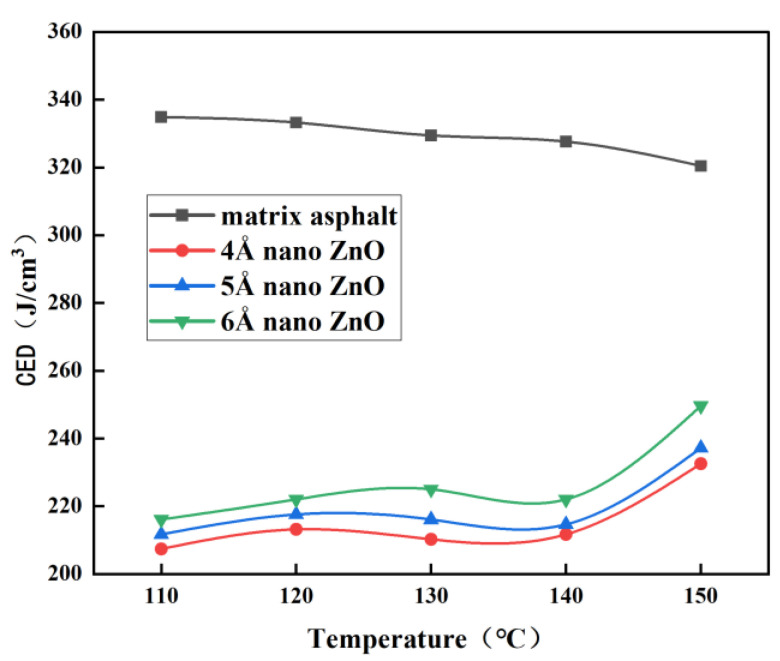
Curves of the cohesive energy density with temperature.

**Figure 9:**
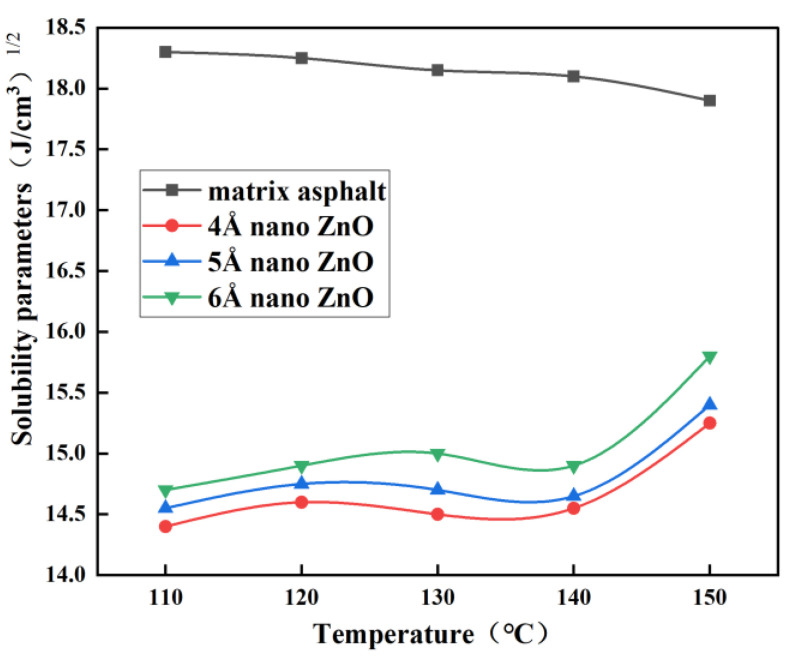
Curves of solubility parameter with temperature.

Firstly, the constructed matrix asphalt model and the nano-ZnO models with different particle sizes were analyzed by NPT molecular dynamics. The simulated conditions are set to a pressure of 1 atm, a time of 500 ps, and different temperatures (110 °C, 120 °C, 130 °C, 140 °C, and 150 °C). The cohesion density and solubility parameters of the four models are calculated according to Equations (1) and (2), and the results are shown in Figure 8 and Figure 9.

From Figure 8 and Figure 9, it can be seen that the cohesive energy density and solubility parameters of the matrix bitumen showed a decreasing trend with increasing temperature from 110 °C to 150 °C, while the cohesive energy density and solubility parameters of nano-ZnO particles fluctuated from 110 °C to 140 °C, but the fluctuations were not significant, while after 140 °C, the cohesive energy density and solubility parameters of nano-ZnO particles increased substantially. Figure 9 also shows that the larger the nano-ZnO diameter particle, the larger the solubility parameter.

**Figure 10:**
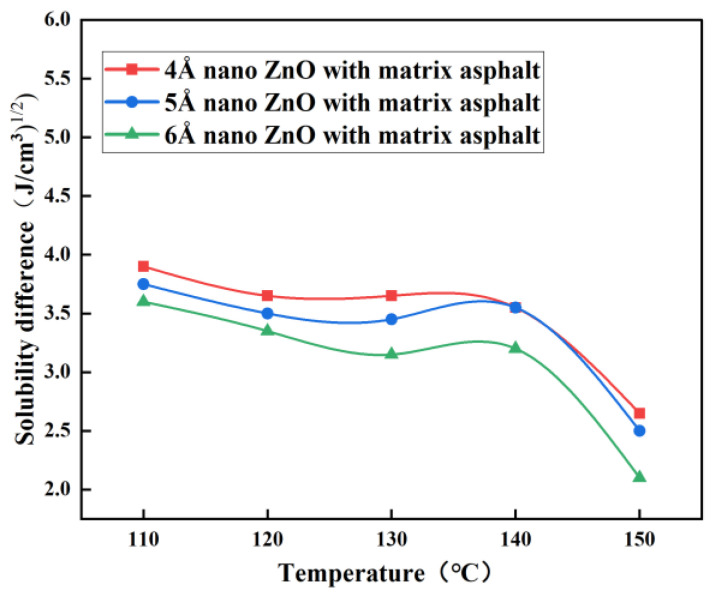
Curves of solubility parameter difference with temperature.

For two different substances, the smaller the difference in solubility parameters, the better the compatibility. To evaluate the compatibility of nano-ZnO with the matrix bitumen, the difference in solubility parameters between nano-ZnO and matrix bitumen was calculated, as shown in Figure 10.

As can be seen from Figure 10, the difference in solubility parameters between nano-ZnO and matrix asphalt tends to decrease from 110 °C to 150 °C as the temperature increases, and the larger the particle size of nano-ZnO, the smaller the difference in solubility parameters between it and the matrix asphalt. At 150 °C, the solubility parameter difference between nano-ZnO particles and matrix asphalt is the smallest, and the compatibility is the best, especially when the particle size of nano-ZnO particles is 6 Å.

### 3.2. Effect of Nano-ZnO on the Interaction Energy of Modified Asphalt

**Figure 11:**
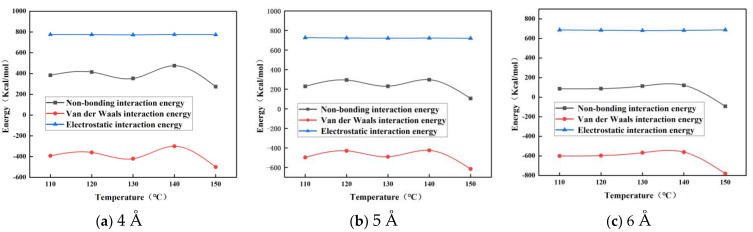
Non-bonding interaction energy of nano-ZnO-modified asphalt at different temperatures.

Molecular dynamics simulations are carried out for different particle size nano-ZnO-modified bitumen models, and the non-bonding energy, van der Waals potential energy, and electrostatic potential energy of different systems at different temperatures are calculated according to Equations (3)–(5), respectively, and the results are shown in Table 2, Table 3 and Table 4 and Figure 11.

As can be seen from Table 2, Table 3 and Table 4 and Figure 11, the electrostatic potential energy of the nano-ZnO particles hardly changes when the temperature increases from 110 °C to 150 °C, but their van der Waals energy, although also changing less in the early temperature rise phase, becomes larger after the temperature rises above 140 °C. The non-bonding energy is the sum of the electrostatic energy and van der Waals energy; therefore, the non-bonding energy of the nano-ZnO particles also changes less in the early stage of temperature rise and more after 140 °C. The change in non-bonding energy is mainly caused by the change in van der Waals energy, and therefore the change in van der Waals energy of the nano-modified bitumen has a greater influence on its compatibility.

**Figure 12:**
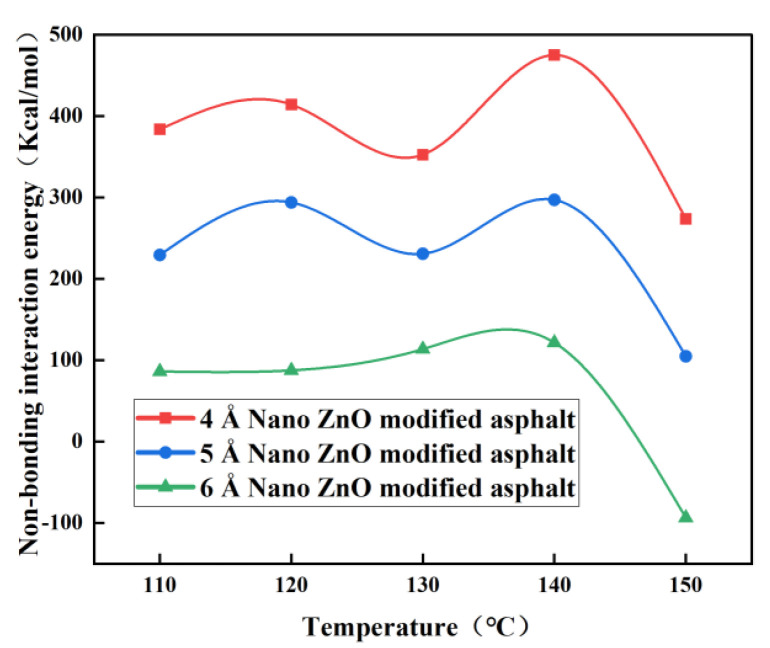
Non-bonding interaction energy of nano-ZnO-modified asphalt with different particle sizes.

The non-bonding interaction energies of the nano-ZnO-modified asphalt with different particle sizes at different temperatures are given in Figure 12. The non-bonding interaction energies of the 4 Å and 5 Å nano-ZnO-modified asphalt at 150 °C are about 273 Kcal/mol and 104 Kcal/mol, where the intermolecular forces are repulsive, and the system is the most stable and compatible because the non-bonding interaction energy is the smallest. However, the non-bonding interaction energy of the 5 Å nano-ZnO-modified asphalt is only about 40% of that of the 4 Å modified asphalt; therefore, the 5 Å nano-ZnO-modified asphalt is more compatible. For the 6 Å nano-ZnO-modified asphalt, the non-bonding energy is about −93 Kcal/mol at 150 °C. At this point, the intermolecular forces are gravitational, and the molecules are not easily separated or destroyed, so the compatibility is best at this temperature compared to other temperatures. It can be seen that for particle sizes of 4 Å, 5 Å, and 6 Å nano-ZnO-modified asphalt, the non-bonding interactions are minimal, i.e., the temperature corresponding to the most stable structure is 150 °C. Therefore, the particle size has little influence on the temperature at which the nano-ZnO-modified asphalt is most structurally stable. At around 150 °C, the nano-ZnO-modified asphalt is more stable, with the smallest non-bonding interactions and the best compatibility.

It can also be seen from Figure 12 that the non-bonding interaction energy of the nano-ZnO-modified asphalt becomes smaller, and the intermolecular forces become smaller and more compatible as the particle size increases from 4 Å to 6 Å at the same temperature. Therefore, at the same temperature and within the particle size range studied, the compatibility of the nano-ZnO-modified asphalt increases with the increasing particle size of nano-ZnO and is best at 6 Å.

### 3.3. Effect of Nano-ZnO on the Mean Square Displacement of Modified Asphalt

**Figure 13:**
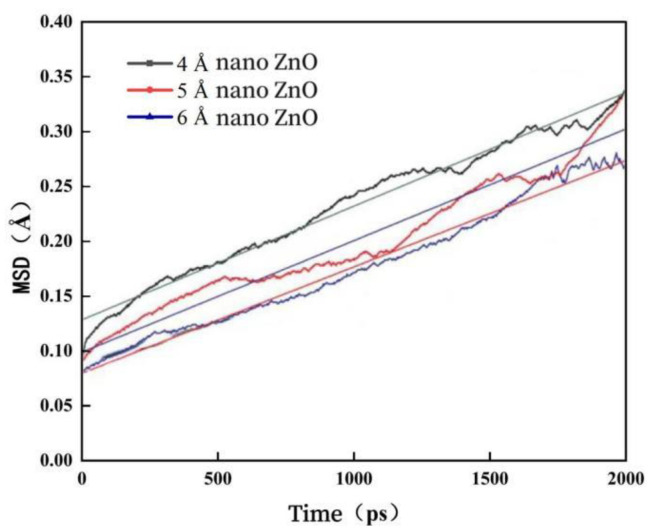
Nano-ZnO mean square displacement curves with time.

In order to investigate the effect of nano-ZnO particle size on the diffusion coefficient of nano-ZnO-modified asphalt, the mean square displacement variation curves of nano-ZnO in different particle size nano-ZnO-modified asphalt systems are calculated, and the results are shown in Figure 13.

As can be seen from Figure 13, the mean square displacement of nano-ZnO is positively correlated with time, with the mean square displacement increasing with time. In addition, when the particle size of nano-ZnO becomes larger, its mean square displacement decreases, and the diffusion coefficient also decreases. In other words, as the particle size of nano-ZnO becomes larger, its ability to diffuse in the bitumen decreases. Therefore, from the perspective of improving the diffusion capacity of nano-ZnO in asphalt, smaller diameter nano-ZnO should be selected.

**Figure 14:**
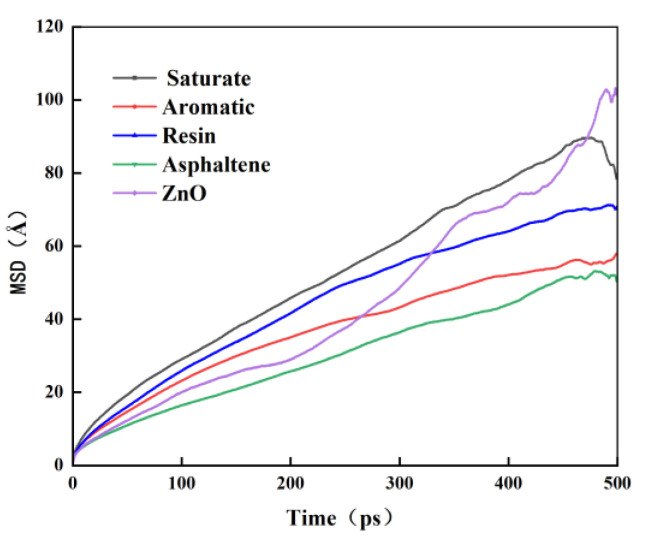
MSD curves for each component of 4 Å nano-ZnO-particle-modified asphalt at 150 °C.

To further investigate the effect of displacement of the components in the nano-ZnO-modified asphalt and their interaction on the compatibility of the modifier with the matrix asphalt, 4 Å of nano-ZnO-modified asphalt is selected and the MSD versus time curves for the four components within the nano-ZnO-modified asphalt system and for nano-ZnO is calculated at 150 °C, as shown in Figure 14.

The saturated fraction is the lightest part of the asphalt and has a strong activity. Asphaltene is heavier in the asphalt and has a weak molecular activity, while the aromatic fraction has fewer bonds and angles and has high molecular activity. As can be seen from Figure 14, the mean square displacement curves of the four components of asphalt in the nano-ZnO-modified asphalt are asphaltene, aromatic fraction, gum, and saturated fraction from the lowest to the highest, which are still the lighter components with stronger activities and the heavier components with weaker activities, so the addition of nano-ZnO modifier does not inhibit the molecular diffusion of the four components in the asphalt, and the interaction between the nano-ZnO modifier and the four components in the asphalt achieves The interaction between the nano-ZnO modifier and the four components of the asphalt achieves the fusion between the nano-ZnO modifier and the four components of the asphalt, thus improving the compatibility between the nano-ZnO modifier and the matrix asphalt.

### 3.4. Effect of Nano-ZnO on the Radial Distribution Function of Four Components of Asphalt

**Figure 15:**
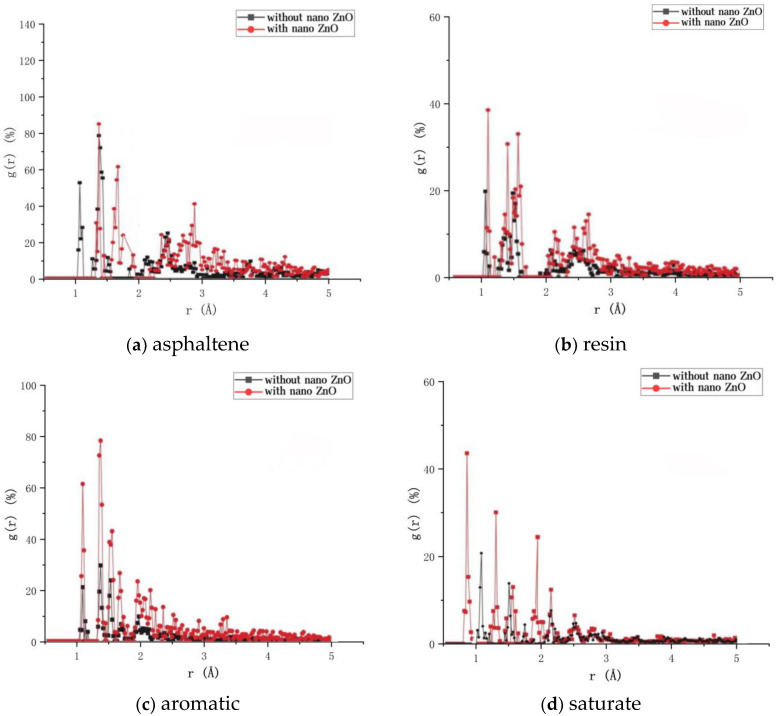
RDF of four components of asphalt before and after the addition of nano-ZnO.

Nano-ZnO with a diameter of 4 Å is taken, and the radial distribution functions of the four components of asphalt before and after the addition of nano-ZnO are calculated to investigate the effect of nano-ZnO on the aggregation behavior of asphalt components and the effect of nano-ZnO modifiers on the molecular structure of asphalt, the results are shown in Figure 15.

The spikes of *g*(*r*) are used to reflect the aggregation of molecules within the asphalt system. An increase in the peak of the first peak indicates denser molecular packing and enhanced orderliness. As can be seen in Figure 15, after the addition of nano-ZnO, the first peak of the four components of the asphalt increases, exhibiting higher and sharper characteristics. Therefore, nano-ZnO makes the asphalt molecules denser and enhances their orderliness. The positions of the first peaks for asphaltene and resin were each shifted to the right by 0.30 Å and 0.08 Å, the aromatic remained unchanged, and the saturate shifted to the left by 0.39 Å. From the shift in the position of the first peak, it can be concluded that the addition of nano-ZnO reduced the aggregation between asphaltene–asphaltene and resin–resin and increased the aggregation of saturate–saturate.

### 3.5. Effect of Nano-ZnO on the Radius of Gyration of Asphalt Four Components

**Figure 16:**
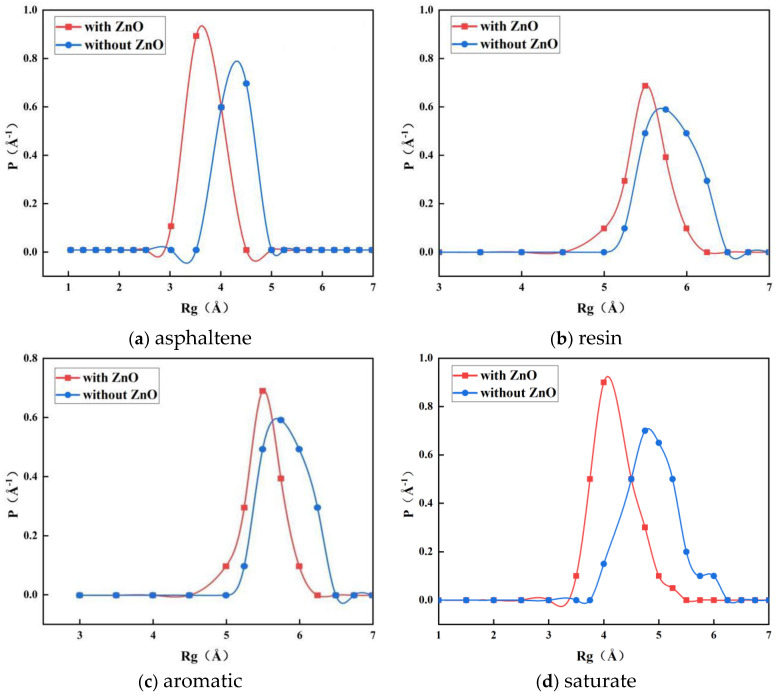
*R_g_* of the four components of asphalt before and after the addition of nano-ZnO.

The radial distribution functions of the four components of the bitumen before and after the addition of four Å ZnO nanoparticles were calculated and are shown in Figure 16.

As can be seen in Figure 16, after the addition of nano-ZnO, the position of the radii of gyration peaks of the four components of asphalt, asphaltene, resin, aromatic, and saturate are shifted to the left. The widths of the *R_g_* peaks of the aromatic and saturate decreased by 0.6 Å and 1 Å. The *R_g_* peaks of the asphaltene and resin remained almost unchanged. When the peak position of the radius of gyration shifts to the left, it indicates an increase in the density of the molecular system, while the decrease in the width of the peak indicates an increase in the branched-chain ductility of the components in the asphalt. Therefore, the addition of nano-ZnO increases the density of the asphalt system and increases the branched chain ductility of the aromatic and saturate, resulting in a denser and more stable asphalt molecule.

## 4. Conclusions

In this study, the influence of nano-ZnO on the compatibility and molecule structure of asphalt was studied by using the MD method. The main findings are as follows:

- The difference in solubility parameters indicates the best compatibility between nano-ZnO and matrix asphalt at 150 °C, especially at a nano-ZnO particle size of 6 Å.
- The particle size of nano-ZnO has little effect on the temperature at which the nano-ZnO-modified asphalt is most structurally stable, with the most stable and compatible system at around 150 °C.
- When the diameter of nano-ZnO becomes larger, its diffusion ability in asphalt is weakened. From the perspective of improving the diffusion ability of nano-ZnO in asphalt, smaller diameter nano-ZnO should be selected.
- The addition of nano-ZnO enhances the intensity of the first peak of the radial distribution function of the four components of the asphalt, the orderliness of the molecules, the ductility of the branched chains of the aromatic and saturated fractions of the asphalt, the addition of nano-ZnO improves the denseness of the molecular structure of the asphalt and makes the asphalt structure more stable.

## Figures and Tables

**Table 1:** Model composition information for nano-ZnO-modified asphalt.

Nano-ZnO Cluster Diameter/Å	Number of Nano-ZnO Clusters/pc	Nano-ZnO Content/%
4 Å	6	4.2981
5 Å	6	4.2861
6 Å	5	4.2933

**Table 2:** Non-bonding energy (Kcal/mol) of nano-ZnO-modified asphalt at different temperatures.

Temperature (°C)	110	120	130	140	150
4 Å ZnO nanoparticle-modified asphalt	383.800	414.372	352.614	475.145	273.801
5 Å ZnO nanoparticle-modified asphalt	229.424	293.941	230.889	297.209	104.934
4 Å ZnO nanoparticle-modified asphalt	86.292	87.644	113.705	121.750	−93.479

**Table 3:** Van der Walls potential energy (Kcal/mol) at different temperatures.

Temperature (°C)	110	120	130	140	150
4 Å ZnO nanoparticle-modified asphalt	−392.209	−360.007	−420.083	−300.408	−500.259
5 Å ZnO nanoparticle-modified asphalt	−497.402	−430.073	−490.781	−425.506	−615.478
4 Å ZnO nanoparticle-modified asphalt	−600.606	−596.358	−567.335	−560.932	−781.241

**Table 4:** Potential energy of electrostatic energy (Kcal/mol) at different temperatures.

Temperature (°C)	110	120	130	140	150
4 Å ZnO nanoparticle-modified asphalt	776.009	774.379	772.697	775.553	774.060
5 Å ZnO nanoparticle-modified asphalt	726.826	724.014	721.670	722.715	720.412
4 Å ZnO nanoparticle-modified asphalt	686.898	684.002	681.040	682.682	687.726

## Data Availability

No new data were created.
